# Complete Genome Sequence of *Halocella* sp. Strain SP3-1, an Extremely Halophilic, Glycoside Hydrolase- and Bacteriocin-Producing Bacterium Isolated from a Salt Evaporation Pond

**DOI:** 10.1128/MRA.01696-18

**Published:** 2019-02-14

**Authors:** Sobroney Heng, Sawannee Sutheeworapong, Peerada Prommeenate, Supapon Cheevadhanarak, Akihiko Kosugi, Patthra Pason, Rattiya Waeonukul, Khanok Ratanakhanokchai, Chakrit Tachaapaikoon

**Affiliations:** a School of Bioresources and Technology, King Mongkut’s University of Technology Thonburi, Bangkok, Thailand; b Pilot Plant Development and Training Institute, King Mongkut’s University of Technology Thonburi, Bangkok, Thailand; c Biochemical Engineering and Pilot Plant Research and Development Unit, National Center for Genetic Engineering and Biotechnology at King Mongkut’s University of Technology Thonburi, Bangkhuntien, Bangkok, Thailand; d Biological Resources and Post-harvest Division, Japan International Research Center for Agricultural Sciences (JIRCAS), Tsukuba, Ibaraki, Japan; University of Maryland School of Medicine

## Abstract

*Halocella* sp. strain SP3-1, a cellulose-degrading bacterium, was isolated from a hypersaline evaporation pond in Thailand. Here, we report the first complete genome sequence of strain SP3-1. This species has a genome size of 4,035,760 bases, and the genome contains several genes encoding cellulose, hemicellulose, starch-degrading enzymes, and bacteriocins.

## Abstract (precis)

*Halocella* sp. strain SP3-1, a cellulose-degrading bacterium, was isolated from a hypersaline evaporation pond in Thailand.

## ANNOUNCEMENT

Halophilic microorganisms thriving in high-salt environments have been reported in bacteria, archaea, and eukarya domains (1). The family *Halanaerobiaceae* comprises five halophilic fermentative genera, including *Halanaerobium*, *Halothermothrix*, *Halocella*, *Halarsenatibacter*, and *Haloincola* (2 – 6). However, only the genome sequences of some species in the genera *Halanaerobium* and *Halothermothrix* (2, 7) have been reported to date. Not much is known about the genus *Halocella,* which was first discovered in 1993, and only one species, Halocella cellulolytica, isolated from the hypersaline lagoons of Lake Sivash (Crimea), has been characterized (4).

The halophilic strain SP3-1 was isolated from soil samples obtained from hypersaline evaporation ponds in Samut Sakhon Province, Thailand. In brief, the soil samples were enriched in basal medium (8) and incubated at 37°C for 7 days, after which the entire process was repeated 6 times using fresh basal medium each time. Individual colonies were isolated from enriched culture using the Hungate roll tube technique (9). The strain SP3-1 was anaerobically cultured at 37°C in basal medium until the late exponential phase, and genomic DNA was extracted from cell culture using the DNeasy blood and tissue kit (Qiagen, Germany). Sequencing libraries were constructed using the SMRTbell template prep kit 1.0 (Pacific Biosciences, Menlo Park, CA). Polymerase reads were trimmed to include only the high-quality region, with a minimum subread length, a minimum polymerase read quality, and a minimum polymerase read length of 500 bp, 0.80, and 100 bp, respectively. Sequencing results contained 170,975 reads totaling 1.4 Gb, with an average read length of 8,343 bases. Sequencing reads were assembled into a complete circular genome with a genome size of 4,035,760 bp, genome coverage of 176×, and G+C content of 35.1%, based on *de novo* assembly using the Hierarchical Genome Assembly Process (HGAP3) (10). Genome annotation was accomplished using Prokka version 1.12b (11) and BLAST2GO (12). The genome contains 3,875 protein-coding sequences, 59 tRNA genes, and 12 rRNA genes.

Phylogenetic analysis of 16S rRNA genes revealed the newly isolated strain SP3-1 is most closely related to Halocella cellulolytica DSM7362, with 92.77% sequence similarity, using the neighbor-joining method (13) via MEGA version 6 (14). *Halocella* sp. SP3-1 showed the ability to produce cellulase, hemicellulose, and amylase, which hydrolyze cellulose, hemicellulose, and starch, respectively, based on the analysis of enzyme activity using a dinitrosalicylic acid (DNS) assay for reducing sugar (15). Twenty-one genes associated with the glycoside hydrolases were discovered in the genome of strain SP3-1 by using Blast2GO (12). Furthermore, the genomic information of strain SP3-1 was used to predict putative bacteriocin-encoding genes, by employing antiSMASH 3.0 (16). Bacteriocins are peptides produced by bacteria that inhibit or kill other related and unrelated microorganisms, making them potentially useful for the food and pharmaceutical industries (17).

### Data availability.

The genome sequence of *Halocella* sp. strain SP3-1 has been deposited in GenBank under the accession number CP032760. The SRA accession number for the raw data is SRR8335321, and the BioProject number is PRJNA493542.
